# A New Isolate *Beauveria bassiana* GxABT-1: Efficacy against *Myzus persicae* and Promising Impact on the Beet Mild Yellow Virus-Aphid Association

**DOI:** 10.3390/insects15090697

**Published:** 2024-09-14

**Authors:** Kenza Dessauvages, Mathilde Scheifler, Frédéric Francis, Ibtissem Ben Fekih

**Affiliations:** 1 Functional and Evolutionary Entomology, Terra, Gembloux Agro-Bio Tech, University of Liege, Passage des Déportés 2, 5030 Gembloux, Belgium; kenza.dessauvages@uliege.be; 2 Evolution and Ecophysiology Group, Functional and Evolutionary Entomology, Terra, Gembloux Agro-Bio Tech, University of Liege, 5030 Gembloux, Belgium; mathilde.scheifler@uliege.be

**Keywords:** aphid-borne virus, beet yellowing, endophyte, hypocrealean fungi, microbial control, multitrophic interactions

## Abstract

**Simple Summary:**

Investigating new microbial control agents to overcome the use of chemical insecticides is of utmost importance to control *Myzus persicae*, the main vector of Beet Mild Yellow Virus (BMYV). Our study explored the efficiency of two *Beauveria bassiana* isolates (GHA and GxABT-1) against *M. persicae* and on the transmission of BMYV. After 8 days (post fungal spray), a mortality rate of more than 90% was registered among treated *M. persicae*. The impact of these fungi as endophytes on the BMYV-*M. persicae* association was assessed by (1) treating sugar beet seeds with fungal suspensions, (2) proving the ability of the fungi to colonize the plant, (3) studying the impact on the aphid’s life cycle and its attraction towards plants, and (4) evaluating the virus load in treated plants. The fungi were able to colonize all parts of the plant, which led to alterations in the aphids’ life cycle and their attractiveness to the plant. Although the fungi were not able to prevent virus transmission, the viral load appears to be reduced. We suggest performing the experiments on a larger scale and using different methods to inoculate the plants to explore whether the efficiency of the tested fungi could be enhanced.

**Abstract:**

Within the context of ecofriendly alternatives to neonicotinoids, we explored the direct and endophytic potential of two *Beauveria bassiana* isolates, GHA from BotaniGard and the new endemic isolate GxABT-1, against the *Sugar Beet Mild Yellow Virus* (*BMYV*)-*Myzus persicae* pathosystem. A mortality rate of 96 and 91% was registered after 8 days of treatment with GHA and Gx-ABT-1, respectively. To assess the endophytic impact, sugar beet seeds were treated, and the ability of the fungi to colonize the plant was assessed and correlated with the aphids’ (1) life cycle, (2) attraction towards the plants, and (3) ability to transmit BMYV. Both fungi colonized the plants, and the GxABT-1 isolate impaired the aphids’ life cycle. *Myzus persicae* were more attracted to leaf discs from non-treated plants than to the fungal-treated ones. Interestingly, when the choice test dealt only with the fungal treatments, aphids were more attracted to leaves from plants harboring Gx-ABT-1 than those with GHA. Moreover, no significant impact was observed for BMYV transmission despite the slight decrease in the viral load in GxABT-1 isolate-treated plants. Our findings constitute a baseline to delve more into the performance of the new endemic isolate *B. bassiana* in other pathosystems using different treatment methods.

## 1. Introduction

The green peach aphid, *Myzus persicae* (Sulzer, 1776), is a highly polyphagous aphid species responsible for the transmission of numerous viruses in various economically important cash crops [1]. *Myzus persicae* is also one of the major pests of sugar beet (*Beta vulgaris* ssp. *vulgaris* L.), being the main vector of sugar beet-associated viruses [2,3,4,5]. The ‘beet polerovirus’ beet mild yellow virus (BYMV) is one of the economically important yellowing viruses causing yield losses of up to 30% [2,6,7]. For many years, neonicotinoid insecticides (i.e., clothianidin, imidacloprid, and thiamethoxam) have been the major chemicals used in seed treatments or foliar applications against aphids and associated beet yellow viruses [4,8]. However, due to the adverse effects on pollinators and the environment, the European Commission took a series of measures since 2018 to restrict the use of some of these chemicals [9,10] and then to completely ban their use in 19 January 2023 (Case C-162/21) [11]. Following these decisions, the beet yellowing disease is likely to become a major threat to the sugar beet industry. Therefore, there is an urgent need to explore innovative and more eco-friendly, sustainable alternative methods of pest control [4,12]. 

Entomopathogenic fungi (EPF) are among the most promising alternatives to neonics, offering multiple ecosystem services. For example, they have a direct entomotoxic effect on insect pests, indirectly promote plant growth, and induce systemic resistance against pathogens and herbivores [13]. Globally distributed, EPF show great potential as effective biocontrol agents [13,14]. However, some of the EPF, including *Beauveria bassiana* (Hypocreales: Cordycipitaceae) and *Metarhizium brunneum* (Hypocreales: Clavicipitaceae), exhibit an endophytic lifestyle by colonizing and living asymptomatically within plant tissues without causing disease in their host [15,16]. Different studies have reported the beneficial impact of their endophytic presence at various levels, including enhancing plant growth, altering the attractiveness of plants to both bio-aggressors and natural enemies, and influencing the development and reproduction of pests, among other factors [17,18]. Finally, both *M. brunneum* and *B. bassiana* exhibit a direct or endophytic effect against insect herbivores [13]. 

Endophytic EPF may also be effective in the management of plant diseases [19]. A few studies have examined their impact on virus transmission by insect vectors, revealing the potential to reduce this transmission, which could be linked to the insect feeding behavior or to systemic resistance to the virus induced by the endophytic EPF [20,21,22]. Research into understanding the mechanisms behind these phenomena is expanding, and many aspects remain poorly understood. To date, no studies have been conducted on the colonization of sugar beet by endophytic EPF and their potential impacts on aphids and the viruses they transmit. In this study, we explored the direct and endophytic effects of an endemic new isolate of *B. bassiana* in the sugar beet-BMYV infected *M. persicae* pathosystem. This exploratory study serves as a foundation for directing subsequent research efforts towards finding more effective eco-friendly microbial agents to decrease beet yellow disease. 

## 2. Materials and Methods

### 2.1. Plants, Insects and Virus

Sugar beet (*Beta vulgaris* ssp. *vulgaris* L.) seeds coated with hymexazol (active ingredient in Tachigaren 70 WS) were provided by the Royal Belgian Institute for Beet Improvement (IRBAB). The treated seeds were sown in seed trays before being transplanted individually into 10 cm diameter pots upon reaching the 2-leaf stage, using a universal potting soil (reference: TERS50, La Plaine Chassart, Fleurus, Belgium). Sugar beet plants were maintained in a growth chamber under controlled conditions at 23 ± 1 °C, 60% RH, and LD 16:8 h.

A BMYV-infected *M. persicae* clone was provided by SESVanderHave (Tienen, Belgium). Aphid rearing was maintained on sugar beet plants in rearing cages (BugDorm 4M4545, MegaView Science Co. Ltd., Taichung, Taiwan). Four- to six-leaf-stage sugar beet plants were added twice per month to maintain the rearing of viruliferous *M. persicae* and for the infection of new plants with BMYV.

### 2.2. Beauveria bassiana Isolates

#### 2.2.1. Origin

Two isolates of *B. bassiana* were used in our study: (i) *B. bassiana* GHA (the active ingredient of the commercial mycoinsecticide BotaniGard ^®^ 22WP (Certis Europe, Brussels, Belgium)) was generated from culture stock kept in a 10% glycerol solution and stored at −80 °C in cryovial tubes at the Laboratory of Functional and Evolutionary Entomology (Gembloux Agro-Bio Tech, Gembloux, Belgium). The isolate was grown on Sabouraud Dextrose Agar (SDA; Darmstadt, Germany) media and incubated at 23 °C for 14 days until fully sporulated; and ii) *B. bassiana* GxABT-1 from soil collected from the hedgerow of a sugar beet field site (50°33′48.9″ N, 4°40′15.2″ E) in Gembloux (Belgium). Three soil cores (around 10 cm of depth) from the plants’ (weeds and/or flowers) rhizosphere were taken randomly using a hand shovel. After each sampling, the tools were rinsed thoroughly with water, followed by 70% ethanol, and then rinsed again with water. The collected samples were stored in small, sealed plastic bags and carried out to the laboratory for fungal isolation following the bait method by Zimmermann [23]. The different samples were sieved (2 mm mesh), and around 20 mL were added to plastic cups (30 mL), with up to 1 mL of tap water added to balance the water content of the samples. Subsequently, three larvae of *Tenebrio molitor* L. (Coleoptera: Tenebrionidae) were added to each cup, which was sealed with perforated lids. The different cups were then incubated in the dark in closed boxes at 21 °C, and a moist paper towel was added to each box to maintain high humidity. The cups were inverted every 2 days over a period of four–five weeks and assessed daily for the presence of dead larvae. Once dead, the collected cadavers were surface-sterilized with 70% ethanol for 10 s, washed three times with ddH_2_O sterile water, and placed on filter paper to absorb the remaining water droplets. The different specimens were incubated in the dark on SDA media supplemented with streptomycin (0.5 mL of 0.6 g mL^−1^), tetracycline (0.5 mL of 0.05 g mL^−1^), and cyclohexamide (1 mL of 0.05 g mL^−1^) at 23 °C and checked for fungal outgrowth for up to 5–8 days. From a total of 27 incubated specimens, only one cadaver showed a fungal outgrowth.

#### 2.2.2. Morphological and Molecular Identification

Fungal features, such as colony color and conidia shape, were used to identify the fungal isolate at the genus level. The conidia shape was observed under a light microscope at 400x magnification. Morphological identification was conducted using the key provided by Humber [24]. 

To pursue fungal identification at the species level, mycelia and conidia cultured on SDA media for 14 days were harvested by scraping off a portion of the fungal culture using a sterile cell spreader. The harvested fungal structures (mycelia and conidia) were then transferred to Eppendorf tubes with sterile steel beads and ground into a fine powder using Retsch-MM 400 (Verder scientific, Haan, Germany). A second grinding was performed after freezing the samples in liquid nitrogen for 30 s. Genomic DNA extraction was performed using a DNeasy Plant Mini kit (Qiagen, Hilden, Germany) following the manufacturer’s instructions. PCR amplification of the ITS1–5.8S–ITS2 region of the rRNA gene cluster [25] was performed using the primer pairs ITS5 (5′-GGAAGTAAAAGTCGTAACAAGG-3′)/ITS4 (5′-TCCTCCGCTTATTGATATGC-3′) [26]. PCR amplification was performed in reaction volumes of 50 µL containing 25 µL of Q5 High-Fidelity 2X Master Mix (New England Biolabs, Hitchin, UK), 2.5 µL of each primer (10 µM), and 5 µL genomic DNA (10 ng/µL). Cycling conditions were denaturation at 98 °C for 3 min, followed by 35 cycles of denaturation at 98 °C for 1 min, annealing at 55 °C for 1 min, and extension at 72 °C for 1 min 30 s, with a final extension at 72 °C for 10 min. PCR amplifications were run on 1% agarose gels (1× TAE buffer) at 110 V for 30 min by electrophoresis, and the products visualized with SYBR Safe (Invitrogen, Carlsbad, CA, USA) in a Biorad Universal Hood II Gel Doc Imaging System. Prior to sequencing, the PCR products were purified with NucleoSpin Gel and a PCR Clean-up Kit (Macherey-Nagel, Düren, Germany) according to the manufacturer’s instructions. Sanger sequencing was performed by Eurofins Genomics (Ebersberg, Germany), and a Basic Local Alignment Search Tool (BLAST) on the National Center for Biotechnology Information (NCBI) database was performed. Afterwards, the gene sequence was aligned with reference sequences obtained from Genbank (Table S1) using MEGA X [27]. The phylogenetic tree of ITS sequences was constructed with the IQ-TREE program [28]. *Beauveria amorpha* was used as the outgroup. Kimura’s two-parameter model (estimated via Akaike Information Criterion using jModelTest v.2.0 [29]) was applied, and maximum likelihood analysis was run with 10,000 ultrafast bootstrap replicates.

### 2.3. Fungal Inoculum Preparation

Fungal inoculum was prepared by harvesting conidia using a sterilized spatula in sterilized 0.03% Tween 80. The fungal solution was then filtered through sterile cheesecloth to remove hyphal debris. Conidia counts was determined using a Burker hemocytometer (Marienfeld, Germany), and the concentration was adjusted to 1 × 10^8^ conidia mL^−1^. Conidial viability was checked by plating 100 µL of 1 × 10^5^ conidia mL^−1^ dilution on SDA plates for 24 h at 23 °C. Germinated and non-germinated conidia were counted, and only those showing >90% viability were used for the bioassays either against aphids or as seed treatments.

### 2.4. Direct Effect of Fungal Treatment on Myzus persicae

A group of ten young adult (fourth instar) aphids was picked up from infested sugar beet plants, placed in a Petri dish (90 mm diameter), and sprayed with 1 mL of a 1 × 10^8^ conidia mL^−1^ solution from a distance of 10 cm. Control aphids were sprayed with 1 mL of a 0.03% Tween 80 solution following the same procedure as the fungal-treated aphids. Each group of treated aphids was then transferred jointly to a sugar beet leaf in a plastic cup containing 1.5% water agar, as described in Ben Fekih et al. [30]. The sugar beet leaf secured in the water agar served as a food source for the aphids during the bioassay. A total of five replicates for each fungal isolate were used, and the different cups were maintained at 23 °C for LD 16:8 h. Dead aphids were recorded daily over a period of 8 days. The cadavers collected daily were immediately surface-sterilized by dipping in 70% ethanol for 10 s, followed by three rinses in sterile distilled water, after which the cadavers were incubated at 23 °C in a Petri dish containing a filter paper moistened with 600 µL of sterile distilled water. The incubated cadavers were checked daily for up to five days for fungal outgrowth.

### 2.5. Seed Sterilization and Treatments

Non-coated sugar beet seed sterilization was conducted following the methods of Rasool et al. [31]. The seeds were immersed in 70% ethanol for 3 min, then in 2% sodium hypochlorite (NaClO) for 10 min, followed by seven rinses with sterile distilled water. Surface-sterilized seeds were air-dried on sterile absorbent filter paper under a laminar flow hood for 40 min. The efficacy of the seed sterilization was assessed by spreading 100 µL of the final rinse water on SDA medium and incubating it for 10 days in darkness at 23 °C. Once dry, the seeds were immersed in a 15 mL solution of 1 × 10^8^ conidia mL^−1^ suspensions of each of the fungal isolates. For the control, surface-sterilized seeds were immersed in a 0.03% Tween 80 solution. The seeds were immersed for each of the treatments and agitated at 100 rpm at 25 ± 2 °C for 24 h. Following the incubation period, the seeds were sown in seed trays and transplanted into individual pots of 10 cm diameter after 2 weeks. Six-leaf stage plants (40 days post inoculation (DPI)) were then used to proceed with the experiments.

### 2.6. Assessment of Endophytic Colonization

**Figure 1:**
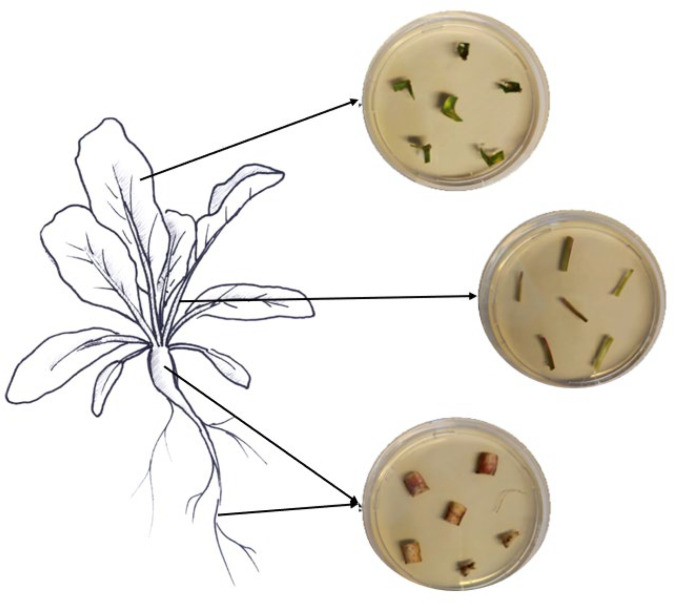
Assessment of fungal endophytic colonization in sugar beet plants.

Samples of roots, hypocotyls, leaves, and petioles were collected from ten randomly chosen treated plants to assess the endophytic colonization of both fungi. From each plant part, six pieces of respective tissues with different sizes were cut with sterile scissors (roots, 1.5 cm; hypocotyls, 2 cm; petioles, 2 cm; leaves, 3 × 3 cm). The various collected plant pieces were subsequently surface-sterilized by immersion for 2 min in 70% and 2% NaClO, followed by three rinses in ddH_2_O sterile water, following Parsa et al. [32]. Sterilization efficacy was assessed as described above. The edges of the surface-sterilized plant tissues were trimmed using sterile scalpels and cut into small pieces (roots, 1 cm; hypocotyls, 0.5 cm; petioles, 0.5 cm; leaves, 0.5 cm). Then, the different plant pieces were partially inserted into SDA media supplemented with the antibiotics mentioned above (Figure 1). The different Petri dishes were incubated at 25 °C in darkness for a period of 14 days. The monitoring of outgrowth mycelia from plant tissues was conducted daily.

### 2.7. Aphid Life Cycle Monitoring on Whole Treated Sugar Beet Plants

Adult aphids were collected from infested sugar beet plants and placed on a sugar beet leaf in a plastic cup containing 1.5% water agar, as described above, to allow for nymph production. The different cups were incubated under controlled conditions at 23 °C for LD 16:8 h. Afterwards, a total of 16 nymphs (24 h-old first instar) produced by these adults were picked up and transferred individually onto the first leaf of treated and non-treated sugar beet plants (of the same age) to assess the effects of the different treatments on their life cycle. The nymphs were confined in clip-cages made from polyurethane foam tubes (internal diameter of 3.2 cm). The clip-cage preparation procedure was inspired by the protocol proposed in Haas et al. [33]. A 2 cm section was cut from the polyurethane foam tube and then incised in the middle to form two discs. A disk of insect-proof film (Nortene^®^) was glued onto one surface of each disc with non-toxic glue. The insect-proof film was made to prevent aphids from escaping while allowing ventilation. Three alligator hair clips were used to bind the two discs of the clip-cage to the leaf. Nymph survival was checked daily for the first 3 days and then every 2 days to monitor their development until adult form and subsequent nymph production. Once the leaves hosting the aphids showed signs of yellowing, the aphids were moved individually to a younger leaf of the same host plant. We ensured a continuous fresh food source for *M. persicae*. An aphid is considered an adult when it produces its first nymph. For this experiment, different parameters were recorded, such as the nymphal development time (time covering all instars period), nymphal mortality (all instars included) occurred during the nymphal development stage, adult fecundity (first instar nymph production), adult longevity (duration of adult stage), and aphid longevity (overall aphid lifespan).

### 2.8. Choice Test

Leaf discs from six-leaf sugar beet plants (treated and non-treated) were used for the choice assays following Fingu-Mabola et al. [34]. One leaf disc was cut from leaves of the same age per plant and placed on filter paper moistened with ddH_2_O in Petri dishes (90 mm in diameter) for 10 min. The filter papers were used to keep the leaf pieces fresh and to reduce volatile emissions due to the cutting [34]. Afterwards, two discs from each treatment (fungal-treated and control) were placed 6 cm apart in a Petri dish filled with moistened filter papers. A total of ten young adult aphids, kept for 2 h without food, were carefully placed in the center of the arena (Petri dish) to assess feeding preference. The different arenas were kept in a ventilated incubator at 23 °C under LD 16:8 h. The total number of aphids on each of the two discs was estimated after 2, 4, and 6 h. Each pairwise treatment was repeated 10 times, with new leaf discs and new aphids (10 arenas).

### 2.9. Virus Transmission

Four viruliferous aphids carrying BMYV were used to infest six-leaf stage plants (treated and non-treated). The different infested plants were, afterwards, covered individually with microperforated plastic to prevent aphid escape. Four days later, the infested plants were sprayed with an acetamiprid-based systemic insecticide (KB^®^ Multisect) to eliminate the aphids. Aphid-free plants were kept in the growth chamber at 23 °C under LD 16:8 h for a period of 4 weeks before proceeding with the virus transmission check experiment. 

After the virus incubation period, leaf pieces from the 36 plants for each treatment were collected. Four leaf discs per plant were collected using a 5 mL Eppendorf tube from leaves of different ages to ensure a similar amount of tissue was gathered from all the plants. These samples were then analyzed with a double antibody sandwich ELISA following the manufacturer’s instructions (Loewe Biochemica GmbH, Sauerlach, Germany). Plates were first coated with 1:250 diluted IgG in coating buffer (pH 9.6) and incubated for 4 h at 37 °C. Leaf samples were ground in grinding bags, diluted to 1:20 in sample buffer (pH 7.4), and duplicates of each sample were placed into 96-well ELISA microplates. The plates were then washed three times with wash buffer (pH 7.2–7.4) before adding 100 µL of the samples. Each sample was duplicated. The plates were incubated overnight at 4 °C. Then, the plates were washed and incubated with AP-conjugate diluted to 1:250 in conjugate buffer (pH 7.4) at 37 °C for a period of 4 h. The plates were washed and incubated with 4-nitrophenylphosphate-di-Na-Salt in substrate buffer (pH 9.8) and left at room temperature for 1–2 h in the dark. Color development was recorded at 415 nm in an ELISA reader (Bio-Rad, iMark Microplate Reader). The absorbances of the two replicates per sample were averaged. Plants were considered virus-infected if the absorbance value was at least twice that of the OD405.

### 2.10. Statistical Analysis

Statistical analyses of the infection bioassays were conducted as follows: The ‘surv’ function from the ‘survival’ [35] package was used to combine survival times and observed events (event: death). Within the same package, the ‘survfit’ function was employed to fit the Kaplan–Meier survival model to these data, estimating the survival function of aphids over time. The ‘ggsurvplot’ function from the ‘survminer’ [36] package was used to graphically visualize the results with confidence intervals.

Data related to plant colonization were analyzed using a binomial generalized linear mixed model (GLMM), where fungal isolates (GHA and GxABT-1) and plant parts (root, petiole, and leaf) were treated as fixed effects. Random effects included plant number and pieces of each plant part.

Analyses of the aphid life cycle were conducted using a GLMM with various distributions tailored to the nature of each variable. Gamma distribution was employed to model the duration of life stages and aphid longevity, while nymphal mortality data were analyzed using binomial distribution (binary data). Additionally, a Poisson distribution was applied to model the number of first instar nymphs produced per aphid over its lifetime. Considering experiments were conducted at two different times, a “time” variable was introduced to the model as a random factor.

Concerning choice test, a GLMM with a Poisson distribution was employed using the “arena” variable as a random factor for repeated measures over time. 

For the BMYV transmission experiments, two complementary approaches were used. Binary data (i.e., infected/non-infected plants) were analyzed using a GLMM, while absorbance data were analyzed using a linear mixed-effects model (LMM). This analysis was conducted to investigate potential differences in viral load. Following the modeling, analyses of variance were conducted to further explore the effects of treatments, and Tukey’s pairwise comparisons were performed when significant differences were observed. All analyses and visualizations of the data were performed using R (version 4.1.3; RStudio Team, 2022).

## 3. Results

### 3.1. Fungal Identification

**Figure 2:**
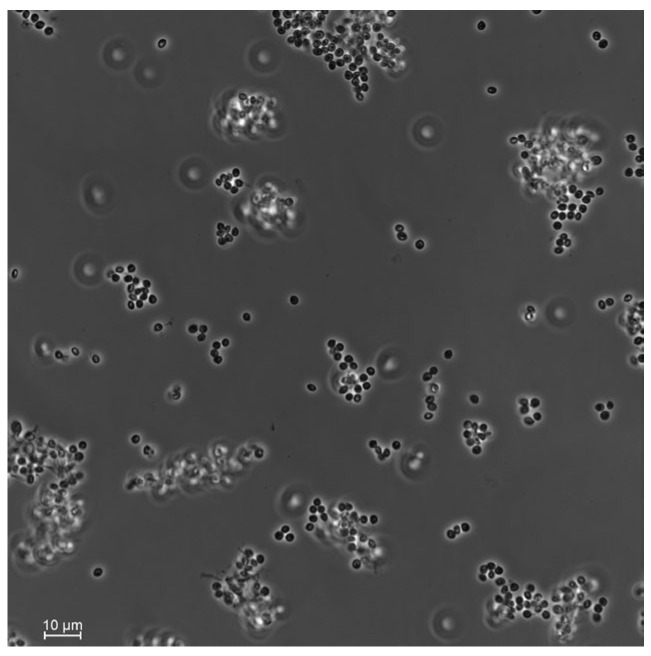
Conidia of the soil-borne fungi *Beauveria bassiana* isolated from the plant rhizosphere in the hedgerow of a sugar beet field. Photos were taken using a Nikon Eclipse Ti2-E inverted automated microscope with a Nikon camera DSQi2.

**Figure 3:**
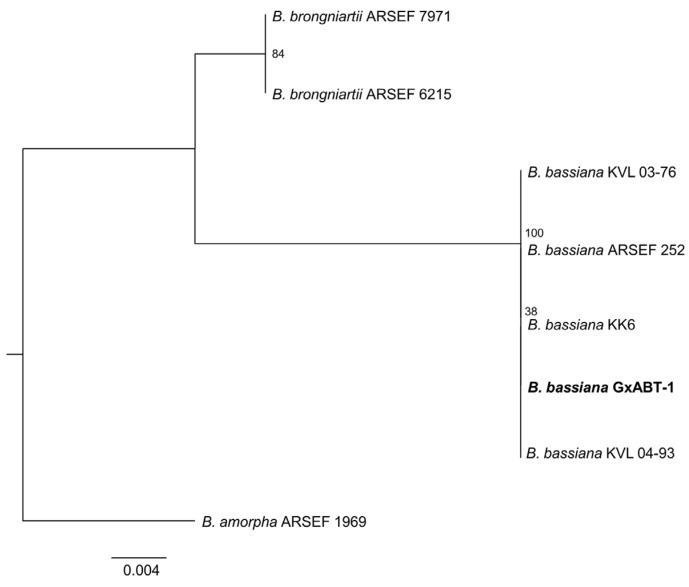
Phylogenetic tree of the ITS1–5.8S–ITS2 region of *Beauveria bassiana* isolates and reference sequences from Genbank (Table S1) analyzed by Maximum Likelihood. Numbers at branches indicate support values, based on 10,000 ultrafast bootstrap replicates. The endemic new isolate of *B. bassiana* isolate is in bold.

From the 27 total *T. molitor* used to bait nine soil samples, only one cadaver showed a typical mycosis caused by Hypocrealean fungi. Conidial dimensions of the endemic fungal isolate were 2.5 ± 0.1 × 2.1 ± 0.2 μm (Figure 2), which is in the range of fungi within the genus *Beauveria* sp. (Hypocreales, Cordycipitaceae). DNA sequencing of the fungal isolate and the high similarity search using NCBI BLAST confirmed it as *B. bassiana*. Phylogenetic analyses constructed with a total of seven ITS sequences of *Beauveria* sp. downloaded from GenBank strongly supported that our fungal isolate was included in the *B. bassiana* clade (Figure 3).

### 3.2. Effect of Beauveria bassiana on Aphid Survival

**Figure 4:**
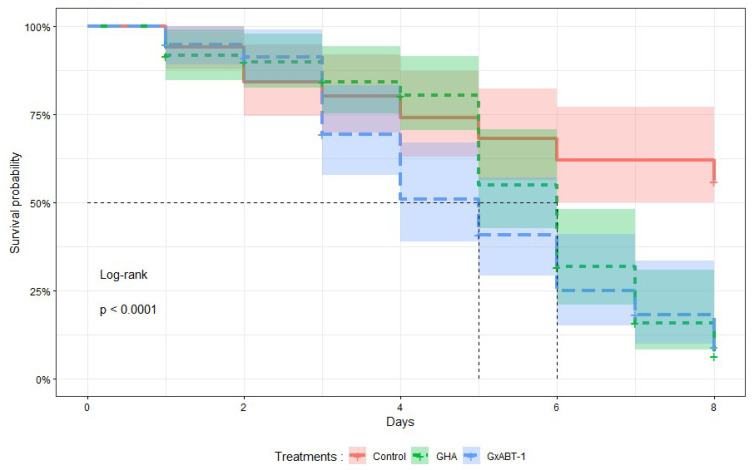
Kaplan–Meier survival curves showing the proportion of surviving treated aphids over time after exposure to two isolates of *Beauveria bassiana,* GHA and GxABT-1. The mean survival time is indicated by the vertical dashed lines for each isolate.

Treatments with both *B. bassiana* isolates significantly reduced the survival time of *M. persicae* individuals (Log-Rank test: χ^2^ = 25, df = 1, *p* < 0.001) compared to those in the control (Figure 4). No significant difference in survival was observed between aphids exposed to both fungal isolates. On average, aphids exposed to *B. bassiana* GHA and GxABT-1 survived 6 and 5 days, respectively. At 8 DPI, on average, 6 ± 4% of aphids treated with GHA isolate survived, 9 ± 5% with GxABT-1 isolate, compared to 56 ± 7% for the control. 

### 3.3. Endophytic Colonization of Sugar Beet Plants

**Figure 5:**
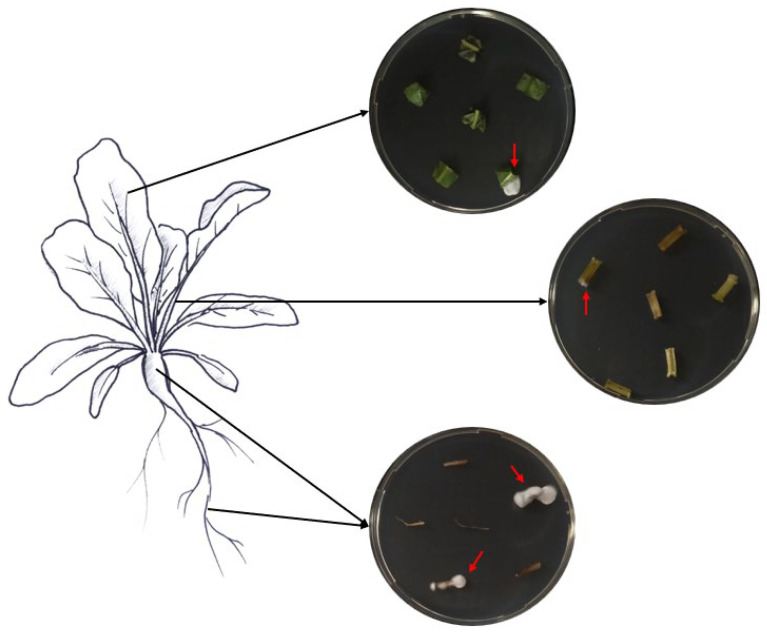
Fungal growth on sugar beet tissues in Petri dishes as a confirmation of endophyte presence.

Inoculation of seeds with *B. bassiana* GHA and GxABT-1 led to the colonization of all tested tissues (i.e., roots, petioles, and leaves) (Figure 5), although we observed varying levels of colonization in the different parts of the sugar beet plants. Since no EPF growth was observed in the control group, it was excluded from the statistical analyses. A significant difference in colonization levels was found between the fungal isolates (GLMM: χ^2^ = 7.51, df = 1, *p* = 0.006), with the GHA isolate being more prevalent in the tissues in general (Table 1). The interaction between plant tissues and fungal isolates was not significant (GLMM: χ^2^ = 5.13, df = 2, *p* = 0.07), suggesting the same colonization pattern for both fungi. Both fungal isolates colonized the roots, petioles, and leaves in the same way, which means that EPF were more commonly found in roots, followed by petioles, and least often in leaves for both isolates.

### 3.4. Indirect Effect on Aphid’s Development and Reproduction 

**Figure 6:**
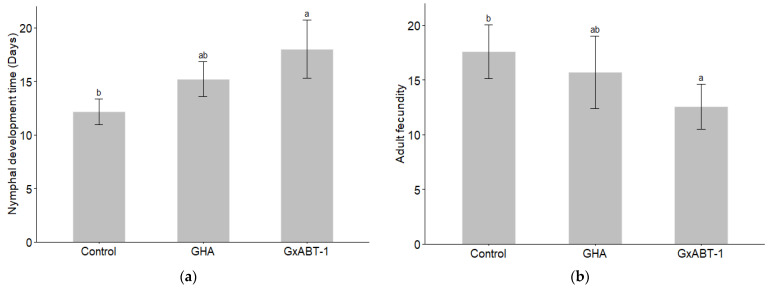
Impact of fungal endophytic colonization of sugar beet plants by *Beauveria bassiana* GHA or GxABT-1 isolates on aphid (**a**) nymphal development time and (**b**) adult fecundity (number of produced first instar nymphs). The significance letters (a, b) indicate statistically different treatments determined by a post-hoc Tukey test.

Significant effects of fungal treatment on both nymphal development time (GLMM: χ^2^ = 9.22, df = 2, *p* = 0.009) and aphid fecundity (GLMM: χ^2^ = 7.41, df = 2, *p* = 0.02) were observed (Table 2). Subsequent post-hoc analysis using Tukey’s test showed that only *B. bassiana* GxABT-1 had a significant impact on both previously mentioned parameters (Table S2). Only *B. bassiana* GxABT-1-treated plants significantly increased the duration of nymphal development stage (*p* = 0.003) by 5.8 ± 3.9 days (from 12.2 ± 1.2 to 18 ± 2.7 days on average) (Figure 6a, Table 2). In addition, the same fungal isolate negatively impacted aphid fecundity by significantly decreasing nymph production (*p* = 0.02): on average, the aphids produced 5.0 ± 4.6 fewer offspring compared to the control treatment (from 17.6 ± 2.5 for the control to 12.6 ± 2.1 for the GxABT-1 isolate) (Figure 6b).

Furthermore, an increased mortality rate of nymphs (all instars included) was observed for both GxABT-1 and GHA (53.3% and 28.6% against 14.3% for control), although the difference was only marginally significant (GLMM: χ^2^ = 5.30, df = 2, *p* = 0.07). The endophytic EPF did not impact adult stage or longevity (Table 2).

### 3.5. Host-Choice Tests

**Figure 7:**
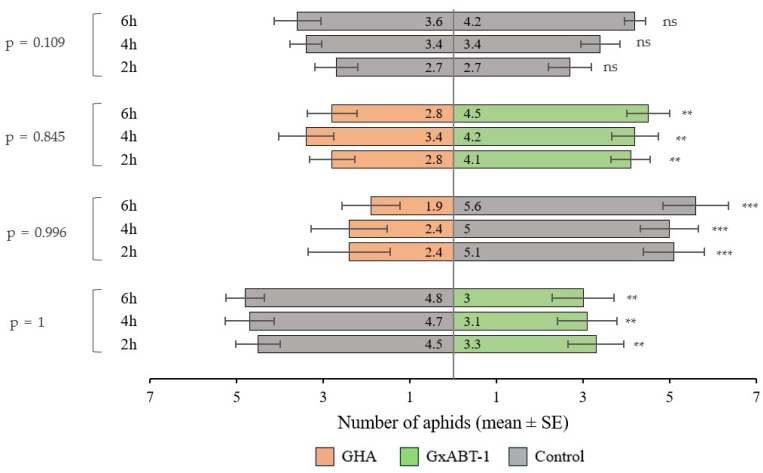
Plant selection by aphids between control and fungal inoculated plants (*Beauveria bassiana* GHA or GxABT-1) during dual-choice tests. Each test involved the release of 10 aphids and observation after 2, 4, and 6 h. The numbers included in the horizontal bars represent the mean number of aphids visting each of the treatements. To the left, the *p*-values represent tests comparing aphid distribution over time. ns, not significant. Asterisks indicate significance levels: ** *p* < 0.01, *** *p* < 0.001.

The preliminary test involving aphid preference between the two control plants showed no bias. The aphids were distributed evenly across the two leaf discs. Aphids showed preferences towards leaf discs from control over those treated with fungi (for both fungal isolates) (Figure 7). They were found more frequently on control discs compared to those treated with *B. bassiana* GHA (GLMM: χ^2^ = 37.20, df = 1, *p* < 0.001) and *B. bassiana* GxABT-1 (GLMM: χ^2^ = 9.10, df = 1, *p* = 0.002). However, when given the choice between leaf discs from both *B. bassiana*-treated plants, aphids showed a preference for the ones treated with *B. bassiana* GxABT-1 over the ones with *B. bassiana* GHA (GLMM: χ^2^ = 6.66, df = 1, *p* = 0.010). The time factor did not influence the choice of aphids among the different treatments (*p*-values > 0.05) (Figure 7).

### 3.6. Impact on Beet Mild Yellow Virus Transmission

**Figure 8:**
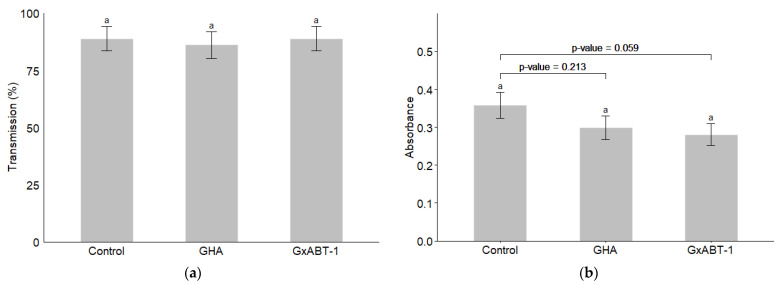
Impact of entomopathogenic fungi colonization on Beet Mild Yellow Virus transmission by *Myzus persicae*. (**a**) Percentage of BMYV-infected plants; (**b**) comparison of the absorbance for inoculated vs. control plants. The significance letter (a) indicates no statistical difference between treatments (post-hoc Tukey test).

Neither of the two *B. bassiana* isolates showed an effect on the transmission of BMYV in terms of infected plants (Figure 8a) (GLMM: χ^2^ = 0.17, df = 2, *p* = 0.918). However, a slight decrease but not significant in the registered absorbance—which reflect the viral load in the tested samples—was observed for the treated plants over those of the controls (LMM: χ^2^ = 5.61, df = 2, *p* = 0.060). Delving deeper with a post-hoc test reveals that this difference lies between the control and the endemic isolate GxABT-1, where a slight decrease in viral load was observed (Tukey: *p* = 0.0595).

## 4. Discussion

Hypocrealean EPF such as *B. bassiana* are extensively studied due to their great interest as microbial control agents against numerous insect pests [14]. The endophytic potential of these EPF has been reported in recent years [37]. For example, *B. bassiana* has shown a direct and endophytic impact against other aphid species, such as *Aphis gossypii,* in addition to *M. persicae* [13]. Furthermore, our study investigated the efficiency of the new endemic isolate *B. bassiana* GxABT-1 against *M. persicae*, both directly and endophytically, and its potential impact on BMYV transmission in sugar beet plants. Investigations exploring the potential of endemic isolates of hypocrealean EPF are of great interest, since by being part of the local biodiversity, they are more adapted than other introduced fungal isolates to control local pests and reduce risks to non-target organisms [38]. In addition, introduced exotic organisms as biocontrol agents might face challenges to adapt to local abiotic and biotic factors, while native fungal isolates can circumvent these difficulties [39].

The direct potential of the two *B. bassiana* fungal isolates, GxABT-1 and GHA, has been assessed in vitro using the spray method. Both fungal isolates were equally virulent against *M. persicae*, with mortality rates of 94 and 91% at 8 DPI and an LT50 of 5 and 6 days, respectively. These results are consistent with other studies evaluating the pathogenicity of *B. bassiana* against *M. persicae* [40,41]. 

Besides their direct efficacy, both fungal isolates showed the ability to colonize, after 40 DPI, all sugar beet plant tissues, with *B. bassiana* GHA being significantly more prevalent in the tissues than GxABT-1. However, interestingly, only plants harboring the new isolate GxABT-1 impacted the aphid life cycle by extending the duration of the nymphal development stage while reducing adult fecundity. Our results are in accordance with Jaber and Araj [42], who reported that the endophytic colonization of *Capsicum annuum* by *B. bassiana* ATCC 74040 caused an extended development time and reduced fecundity of *M. persicae*. However, another study [20] reported an increase in the mortality rate and a reduced fecundity of *M. persicae* feeding on *B. bassiana* GHA-treated tobacco plants. The reduced fecundity was also noted in cotton aphids feeding on *B. bassiana* GHA-inoculated cotton plants [18]. However, the study by Wilberts et al. [43] reported that the treatment of *C. annuum* by *B. bassiana* ARSEF 3097 (ATCC 74040) did not affect *M. persicae* life cycle parameters [43], which contradicts the results by Jaber and Araj [42]. Both studies applied the fungal treatment by root inoculation on the same plant species and using the same *B. bassiana* isolate, but in two different ways, which may explain the differences in efficacy. In the study by Wilberts et al. [43], the roots were rinsed and then soaked in a spore solution, whereas in the study by Jaber and Araj [42], the plants—still in their substrate—were watered with the inoculum. These different results reporting the indirect effect of *B. bassiana* against aphids suggest that the efficiency of the tested endophytic EPF is likely to be dependent on the fungal isolate, plant host, and inoculation method. Following the fungal colonization of plant tissues, the indirect entomotoxic effect might be explained by the production of secondary metabolites, which would impair insect pest fitness [44]. Similarly, the endophytic potential of EPF might involve an upregulation of the genes responsible for the production of phytohormones, which play a role in plant immune responses against herbivores [45]. 

Concerning the choice test experiments, leaf discs from fungal-treated plants were visited significantly less by *M. persicae* than those in the control. Interestingly, aphids facing choices between fungal-treated leaf discs showed that they preferred leaves from GxABT-1-treated plants than those treated with GHA. Our results contradict some other studies that have shown either no effect of the endophytic colonization on the aphids’ choice or a higher attractiveness of colonized plants. For example, Fingu-Mabola et al. [34] reported that *M. persicae* colonies were more attracted to tobacco plant leaf discs inoculated with *B. bassiana* GHA than non-inoculated discs. The same study reported that this attractiveness might be related to the high production of aldehydes by *B. bassiana*-treated plants. It has previously been shown that aldehydes attract aphids [46]. However, in a “whole-plant attractiveness” study, the inoculation with *B. bassiana* ATCC 74040 of *C. annuum* did not impact the choice of *M. persicae* [43]. Similarly, in this context, the endophytic colonization of melon plants by *B. bassiana* (EABb 04/01-Tip and EABb 01/33-Su isolates) did not influence host plant selection [47]. Previous studies have shown that some insect pests other than aphids could detect the presence of EPF in plants and avoid them. For example, the black vine weevil, *Otiorhynchus sulcatus*, avoided grape vines colonized by *B. bassiana* ATCC 74040 when released into a two-choice olfactometer set-up [48]. Similarly, the western tarnished plant bug (*Lygus hesperus*) and the southern green stink bug (*Nezara viridula)* were deterred by cotton plants also colonized by *B. bassiana* GHA under both no-choice and choice assays [49]. These interesting results push towards investigating whether these choices are specific to fungal isolate, host plant, and herbivore associations or whether they are more likely related to the experimental set-up, i.e., whether the test was carried out on plant parts or on whole plants. 

Regarding the virus transmission assay, irrespective of the fungal isolate, the endophytic colonization did not significantly impact the transmission rate of BMYV. Nevertheless, treatment with the GXABT-1 isolate reduced the viral load by 22% compared to the control. However, the difference in viral load between both treatments was only marginally significant (*p* = 0.059), indicating a potential negative effect of *B. bassiana* GxABT-1 on viral transmission. To elucidate the accurate potential of our endemic isolate, it would be interesting to investigate its performance by adopting different methods to treat sugar beet plants (seed treatments, spray, or watering) while ensuring we have heavily BMYV-infected plants that can be used as a source of inoculum for large-scale experiments. Monitoring the evolution of viral load on fungal-treated plants over time and correlating the dynamics of both microbes (virus and fungi) would also help assess the real effect of the endophytic fungi on limiting the virus’ establishment and maintenance in the host. In addition, it would be important to explore the potential of these treatments on non-persistently transmitted potyvirus, one of the most widely distributed pathogens in economically important crops [50]. Investigating the potential of these fungal treatments as well as other microbial agents on different plant pathogens would be beneficial to promote their use in organic [51] and sustainable farming practice. 

## 5. Conclusions

The performance of introduced microbial control agents in a new area is continuously jeopardized by the local (biotic and abiotic) factors of the host environment. Therefore, exploring the agricultural and ecological benefits of endemic microbial control agents is important for overcoming compatibility-related issues. Within this context, we performed our study in which we provided baseline information on the promising potential of the new endemic isolate *B. bassiana*, isolated from a sugar beet agricultural field, against the beet yellowing virus. Interesting results have been reported, but more thorough experiments are still needed at the biological, chemical, and molecular levels to elucidate the performance of this new fungal isolate.

## Figures and Tables

**Table 1:** Percentage (95% confidence intervals, n = 60) of colonized sugar beet tissue pieces (root, petiole, and leaf) at 45 days post seed inoculation with EPF isolates *B. bassiana* GHA and GxABT-1. The significance letters (a, b, c, d, e) indicate statistically different groups determined by a post-hoc test using the estimated marginal means method (emmeans).

Treatments	Root	Petiole	Leaf
*B. bassiana* GHA	60.0% (46.5–72.2) e	30.0% (19.2–43.4) cd	18.3% (9.9–30.9) ab
*B. bassiana* GxABT-1	38.3% (26.4–51.8) de	28.3% (17.8–41.6) bc	13.3% (6.3–25.1) a

**Table 2:** Life cycle parameters (mean ± SE) of *Myzus persicae* fed on sugar beet plants inoculated with *Beauveria bassiana* GHA or GxABT-1 compared to non-inoculated plants. The significance letters (a, b) indicate statistically different groups determined by a post-hoc Tukey test, and asterisks indicate significant differences between the treatments: * *p* < 0.05, ** *p* < 0.01.

Parameters	Control	*B. bassiana* GHA	*B. bassiana* GxABT-1	*p*-Value
	Mean ± SE			
Nymphal mortality (all instars included) (%)	14.3 ± 9.7 a	28.6 ± 12.5 a	53.3 ± 13.3 a	0.071
Nymphal development time (days)	12.2 ± 1.2 b	15.2 ± 1.6 ab	18.0 ± 2.7 a	0.010 **
Adult stage (days)	12.5 ± 1.3 a	12.8 ± 1.6 a	10.0 ± 1.4 a	0.377
Aphid longevity (days)	23.6 ± 1.4 a	23.9 ± 2.3 a	21.6 ± 2.4 a	0.654
Adult fecundity (total)	17.6 ± 2.5 b	15.7 ± 3.3 ab	12.6 ± 2.1 a	0.025 *

## Data Availability

All data and scripts used for the analysis will be provided upon request.

## Supplementary Materials

The following supporting information can be downloaded at: https://www.mdpi.com/article/10.3390/insects15090697/s1, Table S1: Reference sequences from Genbank used to construct *Beauveria* spp. phylogenetic tree; Table S2: Pairwise comparison *p*-values (Tukey) for the two life-cycle variables impacted by the endophytic presence of *B. bassiana* isolates GHA and GxABT-1 (nymphal development time and adult fecundity).
