# Supplementary material for: A New Isolate Beauveria bassiana GxABT-1: Efficacy against Myzus persicae and Promising Impact on the Beet Mild Yellow Virus-Aphid Association
Source: Insects. 2024 Sep 14;15(9):697. doi: 10.3390/insects15090697 (PMC11432153; doi:10.3390/insects15090697)
Supplement: Supplementary file 1 [file insects-15-00697-s001.zip › Table S1.pdf]

Table S1. Reference sequences from Genbank used to construct *Beauveria* spp. phylogenetic tree

**Fungal species**	**Accession number**
B. bassiana KVL 03-76	GU373817.1
B. bassiana KVL 04-93	GU354338.1
B. bassiana ARSEF 252	EU673368.1
B. bassiana KK6	MH259855.1
***B. bassiana* GxABT-1**	**OP592363**
B. brongniartii ARSEF 7971	KJ941127.1
B. brongniartii ARSEF 6215	HQ880781.1
B. amorpha ARSEF 1969	HQ880807.1
