# Supplementary material for: A New Isolate Beauveria bassiana GxABT-1: Efficacy against Myzus persicae and Promising Impact on the Beet Mild Yellow Virus-Aphid Association
Source: Insects. 2024 Sep 14;15(9):697. doi: 10.3390/insects15090697 (PMC11432153; doi:10.3390/insects15090697)
Supplement: Supplementary file 1 [file insects-15-00697-s001.zip › Table S2.pdf]

Table S2. Pairwise comparison p-values (Tukey) for the two life-cycle variables impacted by the endophytic presence of *B. bassiana* isolates GHA and GxABT-1 (nymphal development time and adult fecundity).

Parameters	Control - *B. bassiana* GHA	Control - *B. bassiana* GxABT-1	*B. bassiana* GxABT-1 - *B. bassiana* GHA
Nymphal development time (d)	0.33583	0.00296 **	0.10543
Adult fecundity (total)	0.4978	0.0209 *	0.2194
